# Supplementary material for: Chaotic turnover of rare and abundant species in a strongly interacting model community
Source: Proc Natl Acad Sci U S A. 2024 Mar 4;121(11):e2312822121. doi: 10.1073/pnas.2312822121 (PMC10945849; doi:10.1073/pnas.2312822121)
Supplement: Supplementary file 1 — Appendix 01 (PDF) [file pnas.2312822121.sapp.pdf]

Supplementary material

Chaotic turnover of rare and abundant species in a strongly interacting model community

---

Emil Mallmin[1,*], Arne Traulsen[1], and Silvia De Monte[1,2]

[1]Max Planck Institute for Evolutionary Biology, Plön, Germany
[2]Institut de Biologie de l'ENS (IBENS), Département de Biologie, Ecole Normale Supérieure, CNRS, INSERM, Université PSL, 75005 Paris, France
[*]mallmin@evolbio.mpg.de

Wednesday 29th November, 2023

Contents

S1 Supplementary Figures **1**

S2 Numerical implementation of model simulations **9**

S3 Simplification of the disordered Lotka-Volterra model with mixing **10**

S4 Invasion analysis of few-species equilibria **11**

S5 Solution of intermittency model under unified coloured noise approximation **12**

List of Figures

S1 Sensitive dependence on model parameters . . . 2
S2 Convergence to positive maximum Lyapunov exponent (MLE) . . . 2
S3 Decay of community similarity with time. . . . 3
S4 Scaling of effective community size with richness . . . 3
S5 Robustness of turnover dynamics under model variations . . . 4
S6 Pairwise correlations in species abundances . . . 5
S7 Scaling of AFD power-law exponent with S and λ . . . 5
S8 Phase diagram form adiabatic simulations . . . 6
S9 Variation of community-level observables across the phase diagram . . . 7
S10 Dependence of the effective parameters u, k on S, λ . . . 7
S11 Collective correlation . . . 8
S12 Power-law exponent ν in the chaotic phase . . . 8
S13 Comparison of autocorrelation times . . . 8
S14 Error in approximate formula for $\overline{X}$ . . . 9

S1 Supplementary Figures

As in the main text, unless otherwise stated, reference parameters in simulations are $S = 500, \mu = 0.5, \sigma = 0.3, \lambda = 10^{-8}$.

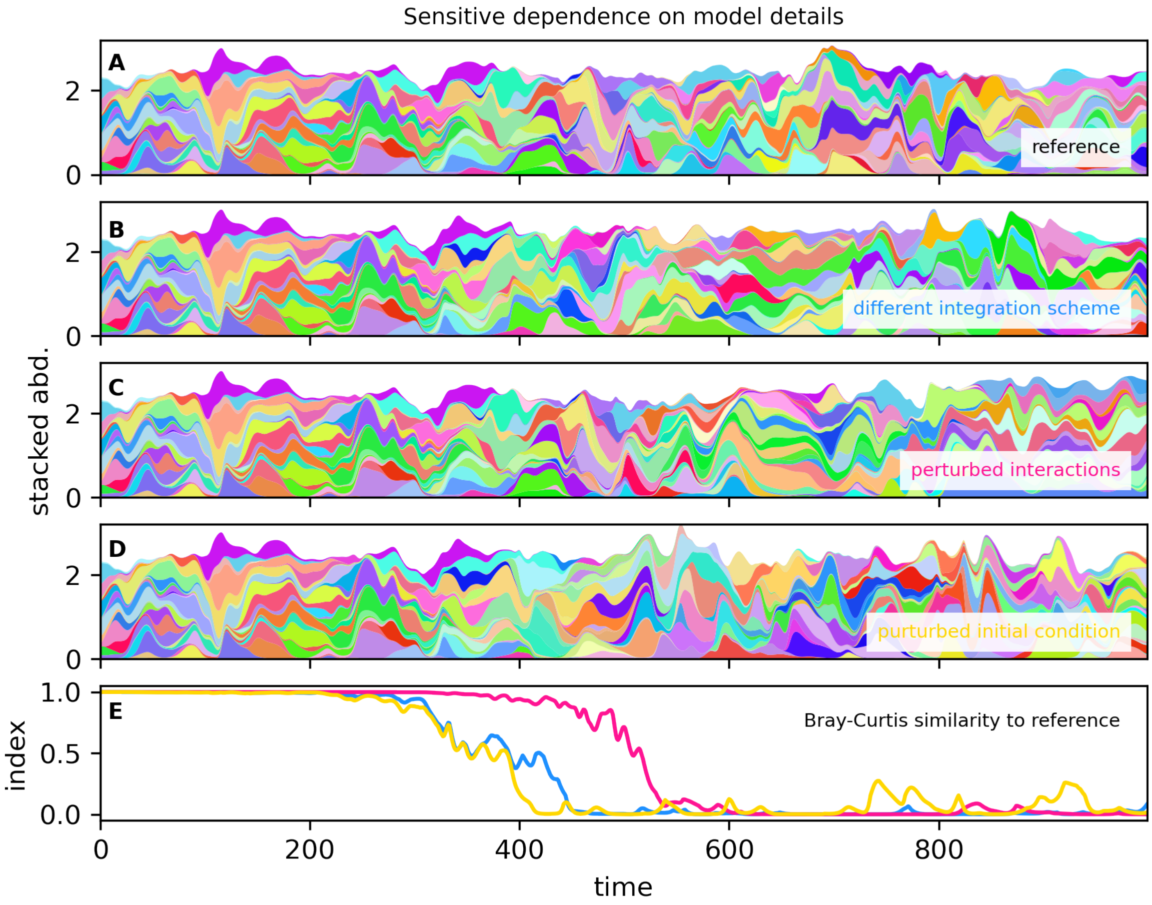

Figure S1: Sensitive dependence on model parameters. A chaotic system exhibits sensitive dependence on initial conditions, and hence also on any model parameters or numerical implementation details that affect the dynamic variables. **A** Reference simulation, showing stacked abundances, similar to Main Text Figure 1A. **B** A change of integration scheme, with respect to the reference; **C** a perturbation of the interaction coefficients by $O(10^{-6})$; **D** a perturbation of the initial abundances by $O(10^{-8})$. **E** Each type of perturbation leads to completely different community composition compared to the reference (measured as Bray-Curtis similarity) after a few hundred time units.

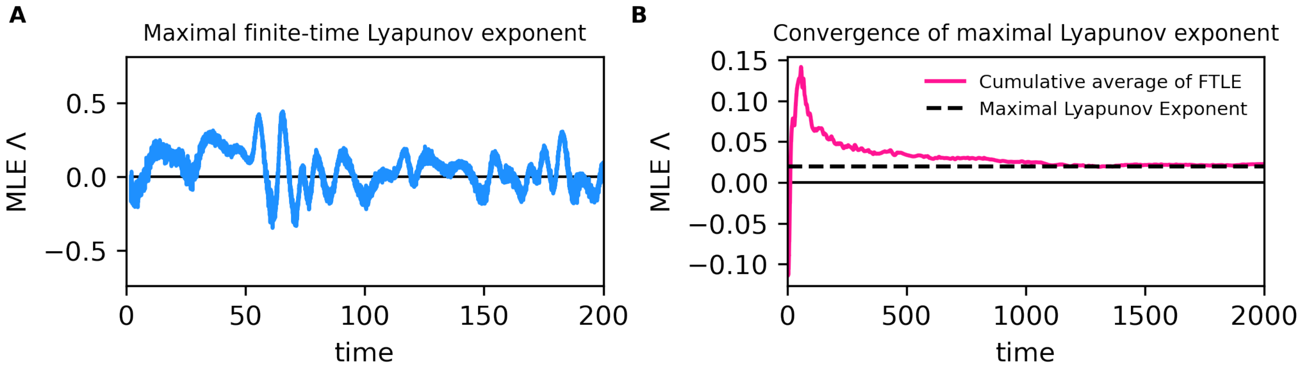

Figure S2: Convergence to positive maximum Lyapunov exponent (MLE). A The dominant finite-time Lyapunov exponent (FTLE) over a few integration time steps ($n = 2$) fluctuates along a trajectory, indicating the alternation of periods of phase-space expansion (boom) and contraction (bust). **B** The cumulative average of the FTLE converges towards a limit that is the maximal Lyapunov exponent. Its positive value (0.02) indicates that the trajectory is chaotic.

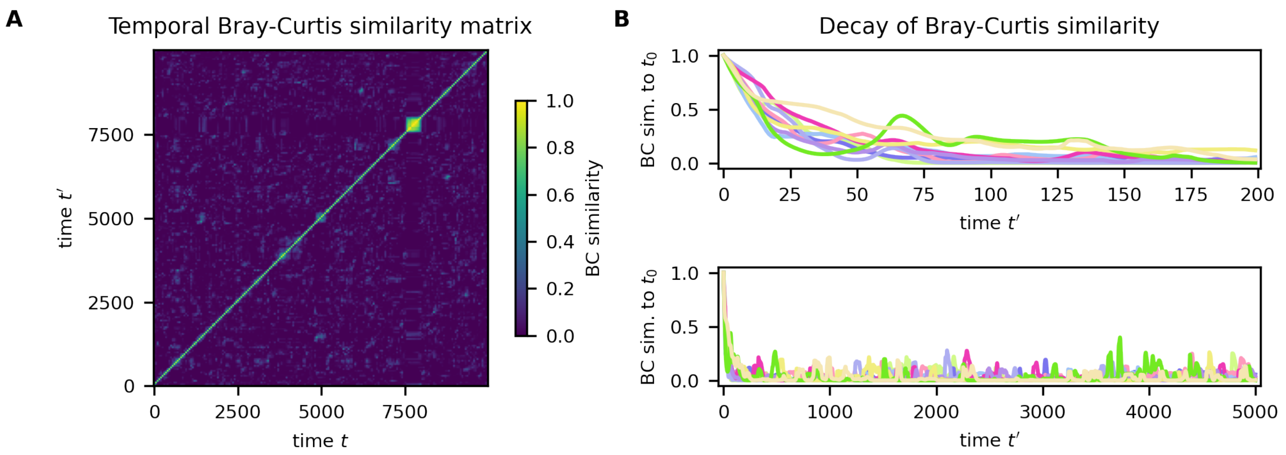

Figure S3: Decay of community similarity with time.. A The temporal similarity matrix $\mathcal{T}$ has elements given by the Bray-Curtis similarity between the abundance vectors at two time points, $\mathcal{T}(t, t') = \mathrm{BC}(\boldsymbol{x}(t), \boldsymbol{x}(t'))$. Because only the diagonal elements are far from zero, and the similarity index is mostly determined by the overlap of dominant species, we conclude that the dominant component is not closely repeated (unless, perhaps, after an exceedingly long time). The aberration around $t \approx t' \approx 8000$ reflects a time when some dominant component persisted for an unusually long time. **B** For a few well-separated time points t (one graph each), we show how $\mathcal{T}(t, t')$ decays over time t' on a timescale of 200 time units (top panel), and how it fluctuates around a small value over a longer time scale of 5000 time units. Thus, community composition decorrelates quickly in time, with some residual low peaks in similarity reflecting that one or a few species will eventually reappear in a dominant community that is otherwise differently composed.

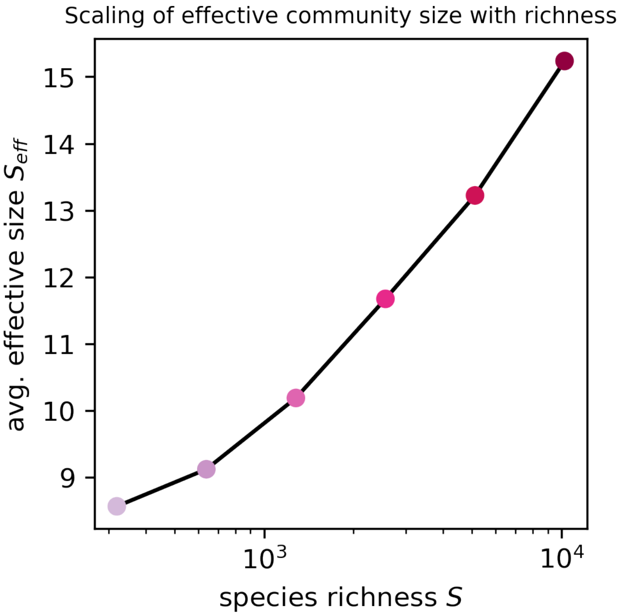

Figure S4: Scaling of effective community size with richness. The time-average of the effective community size, $\overline{S}_{\mathrm{eff}}$ (Main Text Eq. (6)), increases slowly (but super-logarithmically) with the overall richness S. That is, even if we add thousands of new species to the community, the dominant component at given time would just have a species or two more than before.

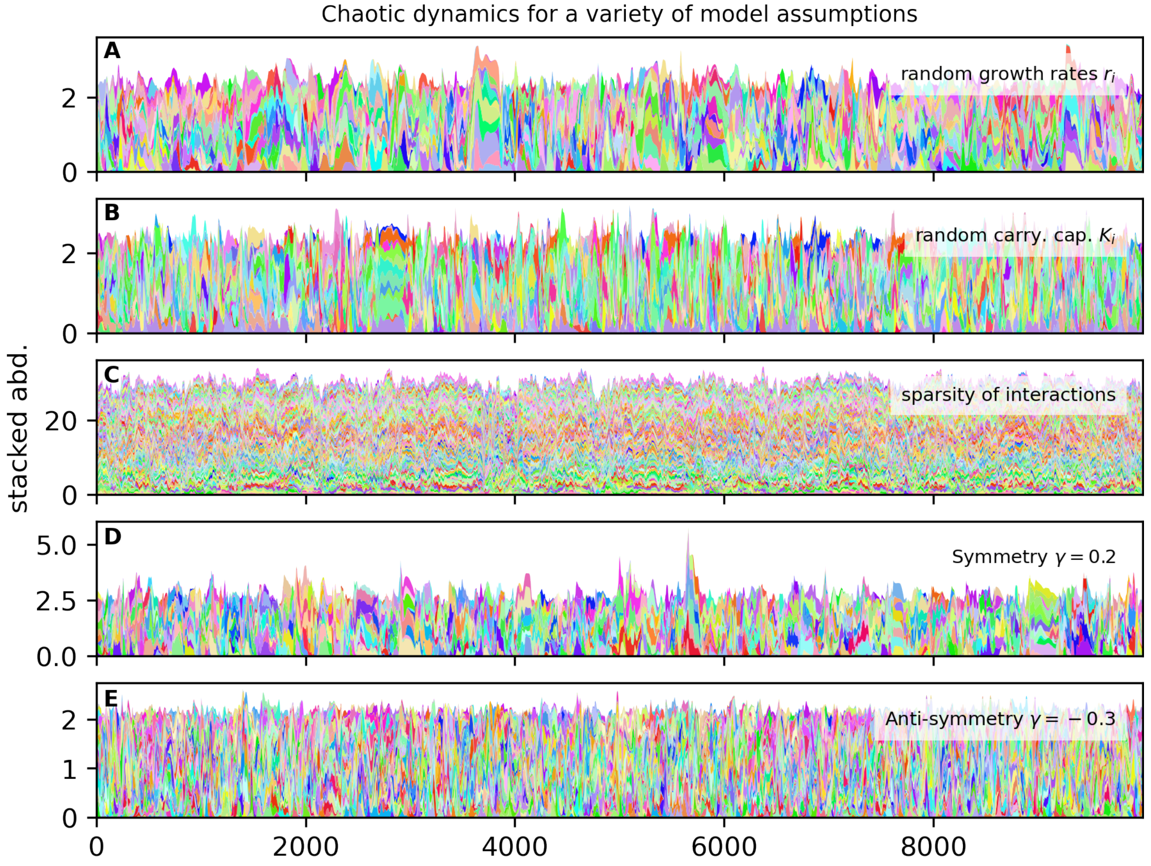

Figure S5: Robustness of turnover dynamics under model variations. We here illustrate that chaotic dynamics is observed even when we relax the simplifying assumptions we made on model parameters in the Main Text; however, we leave a systematic investigation of these generalized scenarios for future work. **A** Non-uniform growth rates: we sample $r_i \sim U(0, 1)$. **B** Non-uniform carrying capacities: $K_i \sim \mathrm{LogNorm}(0, 0.1)$. **C** Sparse interactions: each interaction has a 0.1 chance to be non-zero. **D** Symmetric bias: $\gamma = 0.2$ correlation between diagonally opposed interaction coefficients. **E** Predator-prey bias: $\gamma = -0.3$ correlation between diagonally opposed interaction coefficients.

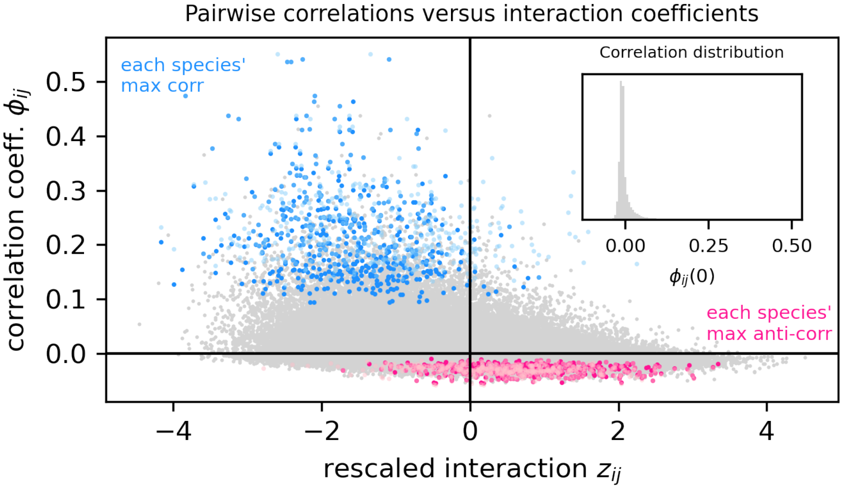

Figure S6: Pairwise correlations in species abundances. While most of the $S(S-1)$ pairs of species do not have meaningful levels of correlation over long times (here 100'000 time units), every species has some other species with which its correlation is substantial and non-spurious. The vertical axis has the correlation coefficient with lag time t_{lag} $\phi_{ij}(t_{\text{lag}})$, and the horizontal axis has the rescaled interaction coefficient $z_{ij} = (\alpha_{ii} - \mu)/\sigma$. The inset show that most zero-lag correlation coefficients are close to zero; all zero-lag correlations are scattered in grey in the main plot. Blue (darker) points shows the values of maximum correlations $\max_j \phi_{ij}(0)$ for every species i; in order to see if correlations are stringer if we optimize over the delay time, we show in light blue $\max_{j,t_{\text{lag}}<200} \phi_{ij}(t_{\text{lag}})$. It is seen that the maximal correlations are around 0.25 in size, and clearly associated with $z_{ij} < 0$, *i.e.* a less-than-averagely negative (even positive) effect of species j on i. Similarly, the extremal anti-correlations (pink for zero time lag, and light pink optimizing over time lag) are associated with $z_{ij} > 0$, *i.e.* a particularly negative effect of j on i.

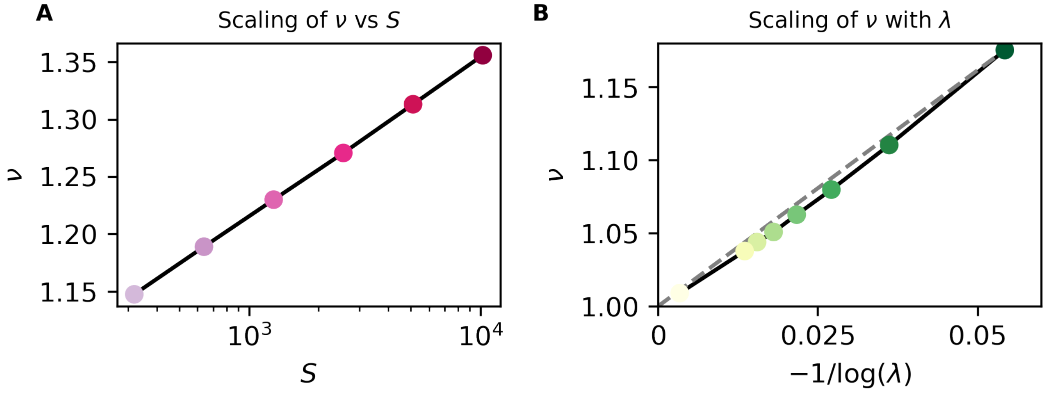

Figure S7: Scaling of AFD power-law exponent with S and λ. From simulations, we have extracted the slope of the power-law section of the abundance fluctuation distribution (AFD) **A** For varying S, we find empirically that the exponent depends linearly on the logarithm of species richness, with coefficients that depend on the system's other parameters: $\nu = \nu_0 + c \log S$, where $\nu_0 = \nu_0(\mu, \sigma, \lambda), c = c(\mu, \sigma, \lambda)$. **B** For varying λ, the exponent appears to follow $\nu = 1 - d/\log \lambda$, where $d = d(\mu, \sigma, S)$. The values of λ are 10 to the power of negative 8, 12, 16, 20, 24, 28, 32, and 128 in order to extrapolate towards zero immigration. The dashed line connects $\nu = 1$ with the value at $\lambda = 10^{-8}$.

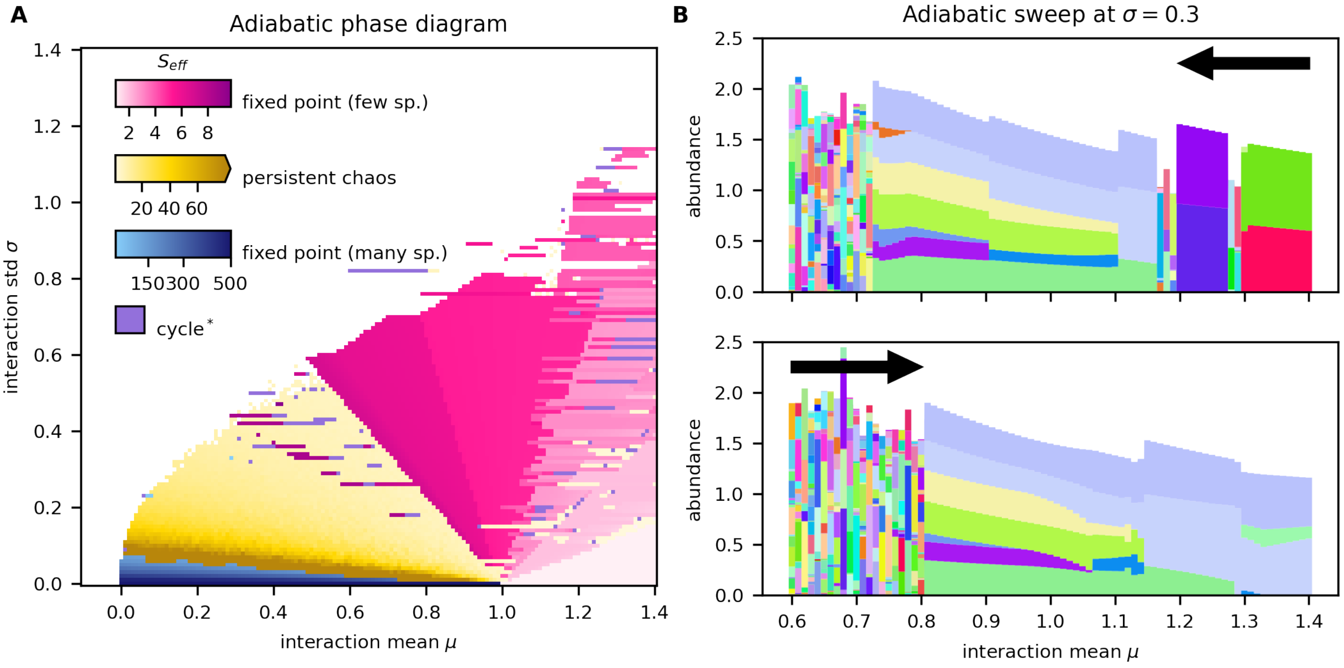

Figure S8: Phase diagram form adiabatic simulations. Adiabatic simulations allow to track, in a numerically efficient fashion, the attractors of the dynamics as model parameters are changed slowly and continuously. To make the interaction statistics μ and σ continuous parameters of the model, we use as interaction matrix $\alpha_{ij}(\mu, \sigma) = \mu + \sigma\zeta_{ij}$ where ζ is a *single, fixed* realization of a standard Gaussian random matrix. **A** For each value of σ, we initialized separate simulation runs starting at $\mu = 1.4$, and let their abundances evolve until an attractor was found. For each run, we then changed μ by small increments $\delta\mu = -0.1$, allowing enough time between each change for the abundances to relax from their previous state. This relaxation would either result in a small perturbation of the previous attractor, or instigate a jump to a different attractor. If a state diverged, the initial abundances for the next value of μ were set as the most recent non-divergent attractor. Thus, each simulation traced a sequence of attractors from $\mu = 1.4 \to -0.1$, corresponding to a horizontal line in the phase diagram. The colour quality reflects the class of the attractor, and the colour gradation indicates the effective community size, revealing the following features: First, we find mostly fixed points in the multiple attractor region. This is because, once a fixed point is converged to, it is "hold on to" until it vanishes or changes stability. If, instead, every simulation at given μ, σ would start from newly sampled initial abundances and interaction matrix, we would find different attractors every time, and the diagram becomes more heterogeneous (compare Main Text Figure 5). Second, clear lines radiate from $(\mu, \sigma) = (1, 0)$ and delineate sectors characterized by the number of high-abundance species coexisting at a fixed-point. In section S4 we show that an invasion analysis predicts such sectors, but not the right scaling of the lines' slope with $\overline{S}_{\text{eff}}$. Third, the jump from fixed-point to chaotic attractors occurs along a sharply defined line. **B** Stacked abundances of the attractor found in an adiabatic sequence $\mu = 1.4 \to 0.6$ (top panel, right to left) and the reverse $0.6 \to 1.4$ (bottom panel, left to right) at $\sigma = 0.3$. One can see sudden jumps to new equilibria involving more (or less) species. In the upper panel, reading right to left, a three-species equilibrium is found at $\mu = 1.15$, which jumps to a 6-species equilibrium by the invasion of three more species at $\mu = 1.11$; another two species displace one of the previous at $\mu = 0.9$; and at $\mu = 0.72$ a sudden jump onto a chaotic attractor occurs. Reversing the adiabatic protocol, the transition from chaos to fixed point occurs only at $\mu = 0.81$, and the sequence of equilibria is not identical to the forward direction (hysteresis). A systematic investigation of the multiple attractor phase and the transition to the chaotic phase is left for future work.

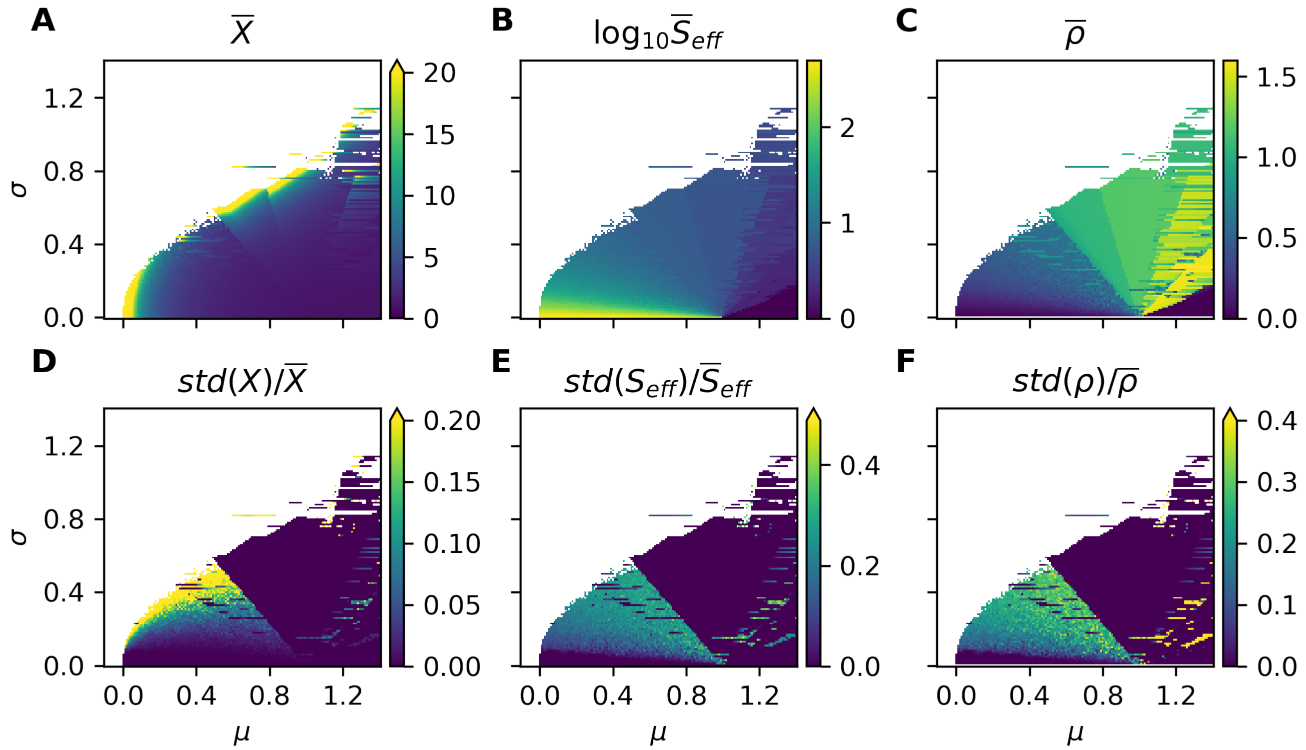

Figure S9: Variation of community-level observables across the phase diagram. For the community-level observables in Main Text Eq. (11) we show: **A–C** their time-averaged values; **D–F** their relative relative fluctuations. The data comes from the adiabatic simulation detailed in the caption to Figure S8. An arrow on the end of the colour bar implies the range has been capped for clarity.

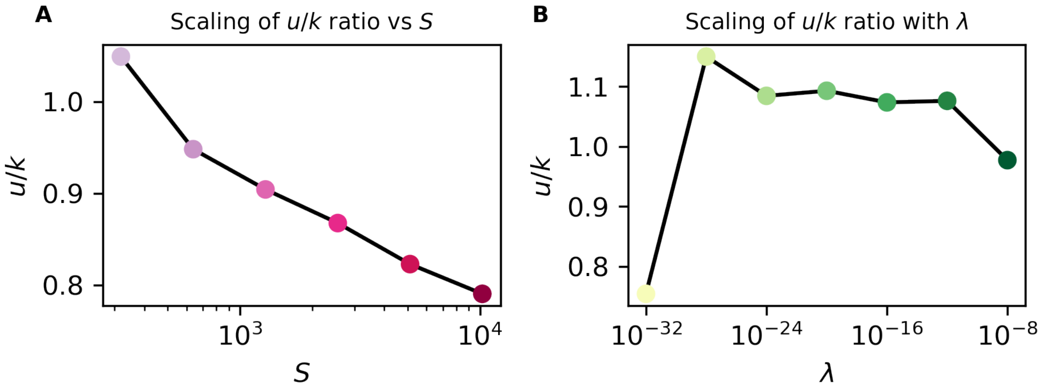

Figure S10: Dependence of the effective parameters u, k on S, λ. The empirical, approximate relationship $u \propto k$, found across the range of μ, σ in the chaotic phase, has a proportionality constant that depends relatively weakly on S and λ.

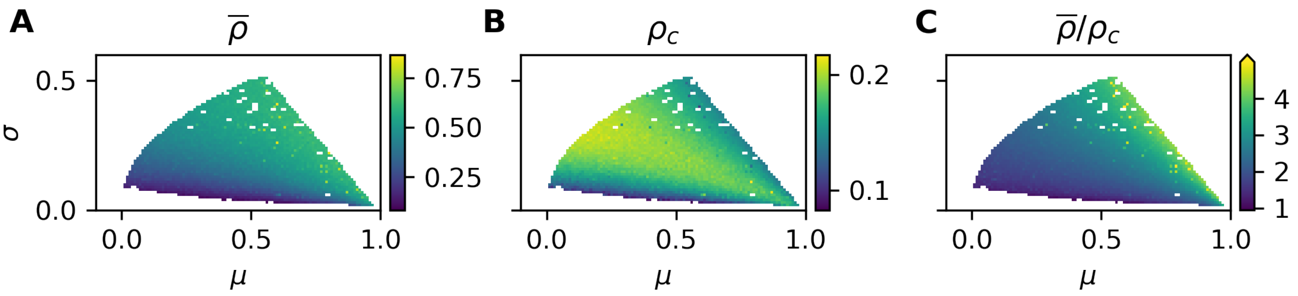

Figure S11: Collective correlation. Within the bounds to the chaotic phase indicated in Figure S8A, we have run a long simulation for each parameter point with random initial condition and interaction matrix realization. Statistics were recorded for persistently chaotic trajectories; non-chaotic trajectories were discarded, and the parameter point rerun to obtain a long chaotic trajectory, up to five times, else the point was omitted (chaos probability was shown in Main Text Figure 5). **A** The collective correlation. **B** The critical value of the collective correlation as defined by Main Text Eq. (13). **C** The ratio $\overline{\rho}/\rho_c$ tends towards 1 at the boundary to the equilibrium phase. Note that $\overline{\rho}$ changes continuously across this boundary (Figure S9C). The arrow at the upper end of the colour bar implies the range has been capped for clarity.

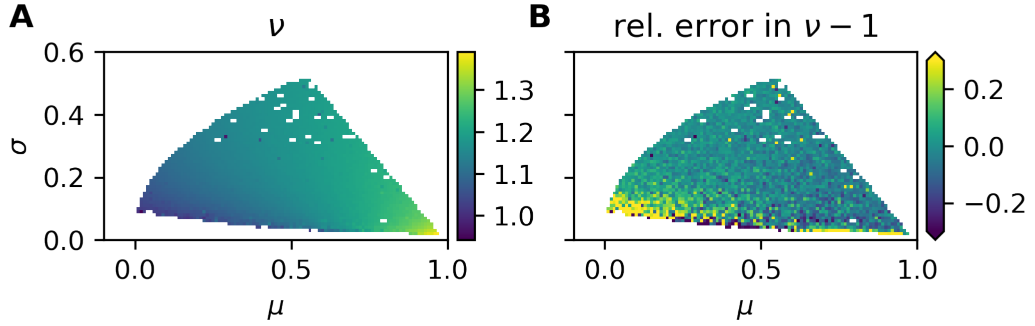

Figure S12: Power-law exponent ν in the chaotic phase. **A** Variation of the AFD power-law exponent across the chaotic phase. Apart from outliers, we find an exponent larger than one. **B** To test the accuracy of the focal-species model in predicting the exponent, we measure the relative error in $\delta\nu - 1$ (since we expect $\nu > 1$) with respect to the value obtained from simulations of the disordered Lotka-Volterra model. Data from the simulations described in Figure S11. The arrow at the upper end of the colour bar implies the range has been capped for clarity.

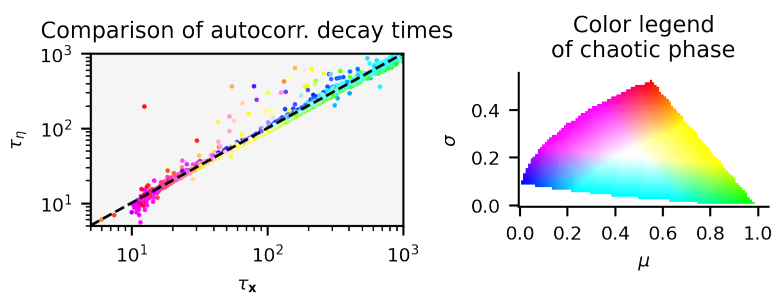

Figure S13: Comparison of autocorrelation times. We compare the autocorrelation time τ_x of the abundance vector $\boldsymbol{x}$ and the autocorrelation time τ_η of the effective noise η. These two parameters are obtained by the exponential fit $e^{-t/\tau}$ applied to the respective autocorrelation functions. Across the chaotic phase, these to timescale are quantitatively close, for reasons explained in Main TextAppendix E.

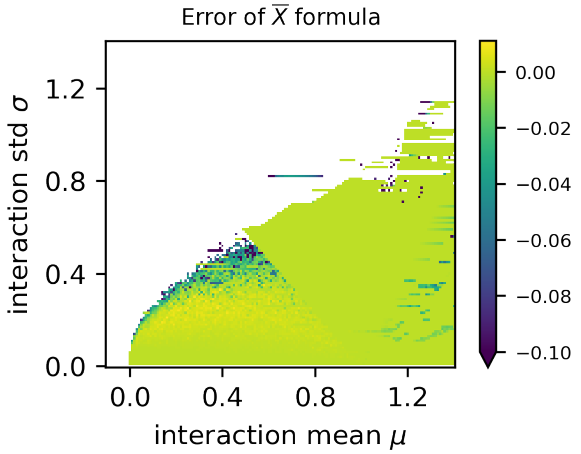

Figure S14: Error in approximate formula for $\overline{X}$. Here is shown that the approximate Main Text Eq. (11) generally gives an accurate prediction (small relative error) of $\overline{X}$ compared to its simulated value (adiabatic data; Figure S8), if given the values of $\overline{S}_{\text{eff}}$ and $\overline{\rho}$ from the same simulation. Except for close the the divergent phase, the error is within $\pm 2\%$. Since the approximations involved in deriving the formula amount to neglecting fluctuations, it is expected to most accurate when fluctuations are small (compare Figure S9); in particular, at fixed-points of the dynamics it becomes exact up to an amount proportional to the negligibly small immigration rate. An arrow on the end of the colour bar implies the range has been capped for clarity.

S2 Numerical implementation of model simulations

All numerical procedures of this work were carried out in `python` using the `numpy` and `scipy` packages. To simulate the disordered Lotka-Volterra model, we have opted for a fixed time integration with a small time step of $\Delta t = 0.01$. By default, we have used the logarithmic integration scheme defined below, whose implementation we have validated against a 'logistic' scheme (Figure S1) and the standard ODE solver of `scipy` using RK45. For simulations of the stochastic differential equation of the focal-species model we have simulated the coloured noise (Ornstein-Uhlenbeck process) under a Euler-Mayurama scheme with $\Delta t = 0.01$, and the abundance dynamics under the logistic scheme with the same time step.

In phase diagram simulations, we have used an expedient numerical heuristic to classify the long-term dynamics of trajectories, which we have validated against visual inspection, and measurement of the maximal Lyapunov exponent for a sample set of trajectories. First, if trajectories diverged, they tended to do so early in the simulation. Otherwise, after a transient interval $[t_0, t_1]$ of fixed duration, we hypothesised that the following time interval $[t_1, t_2]$ would contain stationary dynamics, assuming $t_2 - t_1$ to be longer than any periodicity of the dynamics, if present. We then counted how many times n within this interval the abundance vector $\boldsymbol{x}(t)$ crossed a threshold of $1-\varepsilon$ similarity to the final vector $\boldsymbol{x}(t_2)$, where ε is a small tolerance, and the similarity metric is $d(\boldsymbol{x}, \boldsymbol{y}) = 1 - ||\boldsymbol{x} - \boldsymbol{y}||/(x^2 + y^2)$. If $n = 0$, then abundances were constant in the interval and we assume a stable fixed point has been reached; if $n = 1$, then the final composition was one not seen before in the interval, which we classified as chaos; if $n > 1$ then periodicity or quasi-periodic. For trajectories classified as chaotic, a subsequent long time interval $[t2, t3]$ was simulated and used to gather relevant statistics; finally, the n-classification was applied to a final interval $[t3, t4]$ to ascertain that chaotic dynamics were not lost during the previous interval of measurements.

Definition of integration schemes

We consider the numerical integration of

$$\dot{x}_i(t) = r_i x_i(t)(g_i(t) - x_i(t)), \quad g_i(t) = 1 - \sum_{j(\neq i)} \alpha_{ij} x_j(t). \tag{S1}$$

Because abundances may become exponentially small, yet eventually recover, it is potentially problematic if numerical error can cause zero or negative abundances. To avoid this issue, we can consider the exact identity

$$x_i(t+\Delta t) = x_i(t)\exp\left(\int_t^{t+\Delta t}(g_i(t') - x_i(t'))dt'\right). \tag{S2}$$

Since the exponential is always positive, any numerical integration scheme $x_i(n\Delta t) \to x_i[n]$ based on approximating the integral will preserve positivity of abundances. For a Δt much smaller than the turnover timescale of the dominant community, the integrand can be treated as approximately constant, yielding the scheme

$$x_i[n+1] = x_i[n]\exp\left(\tilde{r}_i - \sum_j \tilde{\alpha}_{ij}x_j[n]\right), \tag{S3}$$

where, for compactness, we have defined $\tilde{r}_i = r_i\Delta t$, and $\tilde{\alpha}_{ij} = \tilde{r}_i\alpha_{ij}$ $(i \neq j)$, $\tilde{\alpha}_{ii} = \tilde{r}_i$. This 'logarithmic' scheme is equivalent to applying a standard Euler scheme to the evolution of log-abundances, $y_i(t) = \ln x_i(t)$. Indeed, any integration scheme applied to log-abundances will preserve positivity.

Another approach is based on the formal solution

$$x_i(t+\Delta t) = x_i(t)\cdot\frac{G_i(t+\Delta t|t)}{1 + r_i x_i(t)\int_t^{t+\Delta t}G_i(t'|t)dt'}, \quad G_i(t'|t) = e^{r_i\int_t^{t'}g_i(s)ds} \tag{S4}$$

The difference to Eq. (S2) is that the right-hand-side does not explicitly depend on x_i. Any approximation of the integral in G_i will preserve positivity of abundances. Choosing $G_i(t'|t) \approx \exp(r_i g_i(t)(t'-t))$ for $t'-t$ small, then performing the integral in the denominator of Eq. (S4), we obtain

$$x_i(t+\Delta t) \approx \frac{g_i(t)x_i(t)}{g_i(t)e^{-r_i g_i(t)\Delta t} + x_i(t)(1 - e^{-r_i g_i(t)\Delta t})}, \tag{S5}$$

The resulting 'logistic' integration scheme is thus (after some rearrangements)

$$x_i[n+1] = g_i[n]\times\frac{x_i[n]}{x_i[n] + (g_i[n] - x_i[n])e^{-r_i g_i[n]\Delta t}}, \quad g_i[n] = 1 - \sum_{j(\neq i)}\alpha_{ij}x_j[n]. \tag{S6}$$

This scheme was proposed by Jules Fraboul [1].

To either integration scheme we can add a term $+\lambda\Delta t$ for the immigration.

S3 Simplification of the disordered Lotka-Volterra model with mixing

We consider a well-mixed volume V containing S species with instantaneous absolute abundance $N_i(t)$, and constant nominal carrying capacities K_i and growth rates R_i. The growth dynamics follows the standard Lotka-Volterra form. We add the effect of mixing with an external environment containing the same set of species but at abundances $N_i^{\text{ext}}(t)$: a fraction Λ per unit time of the volume V is exchanged with an equal volume from the external environment that gets instantaneously mixed in with the volume V. In total, the dynamics of the abundances is

$$\dot{N}_i(t) = R_i N_i(t)\left(1 - \frac{N_i(t) + \sum_{j(\neq i)}\beta_{ij}N_j(t)}{K_i}\right) + \Lambda(N_i^{\text{ext}}(t) - N_i(t)). \tag{S7}$$

Note that if we let $\Lambda \gg \max_i R_i$, we will force $N_i(t) \approx N_i^{\text{ext}}(t)$. Instead, we consider the slow-mixing scenario $\Lambda \ll \min R_i$, since our purpose in adding mixing is mainly to prevent extinction of rare species. We introduce

rescaled parameters

$$r_i = R_i - \Lambda \approx R_i, \tag{S8}$$

$$K_i' = K_i \left(1 - \frac{\Lambda}{r_i}\right) \approx K_i, \tag{S9}$$

$$\alpha_{ij} = \frac{\beta_{ij} K_j'}{K_i'}, \tag{S10}$$

$$\lambda_i(t) = \Lambda \frac{N_i^{\text{ext}}(t)}{K_i'}, \tag{S11}$$

$$x_i(t) = \frac{N_i(t)}{K_i'}, \tag{S12}$$

so that

$$\dot{x}_i(t) = r_i x_i(t) \left(1 - x_i(t) + \sum_{j(\neq i)} \alpha_{ij} x_j(t)\right) + \lambda_i(t). \tag{S13}$$

Since the mixing occurs slowly, we suppose that it is justified to replace N_i^{ext} with its time-average $\overline{N_i}^{\text{ext}}$. A parsimonious distribution for these abundances is that they result from effectively independent species (in particular in the chaotic phase; see main text) constrained by a roughly constant total community biomass N^* independent of the number of species:

$$\overline{N_i}^{\text{ext}} = N^* \frac{K_i}{\sum_j K_j}. \tag{S14}$$

Introducing $\tilde{\lambda} = \Lambda N^*/K^*$ with $K^* = S^{-1} \sum_j K_j$ we have

$$\lambda_i(t) \equiv \lambda = \frac{\tilde{\lambda}}{S}. \tag{S15}$$

Upon the simplifying assumptions $r_i \equiv 1$ and Gaussian distribution of α_{ij} (resulting from a combination of some distribution of K_i and β_{ij}; see [2]), we obtain the model of the main article.

S4 Invasion analysis of few-species equilibria

Let $\mathcal{R}(t), \mathcal{D}(t)$ be the sets of rare and dominant species. The dynamics of a rare species $i \in \mathcal{R}(t)$ that successfully invades is

$$\dot{x}_i \approx x_i \left(1 - \sum_{j \in \mathcal{D}} \alpha_{ij} x_j\right) := \gamma_i x_i, \tag{S16}$$

while while $\lambda \ll x_i \ll 1$. The growth rate γ_i must be (mostly) positive in the time interval where invasion occurs. Thus, no rare species is expected to be able to invade the dominant component while

$$\gamma_{\max} := \max_{i \in \mathcal{R}} \gamma_i < 0. \tag{S17}$$

Suppose then that the $|\mathcal{D}| \approx \overline{S}_{\text{eff}}$ dominant species are in a few-species equilibrium that would be stable but for a potential invasion. We approximate the abundances of dominant species as equal (we expect them at least to be of the same order) and recall that they make up the overwhelming share of the total abundance. Thus, $x_j \approx \overline{X}/\overline{S}_{\text{eff}}$

for $j \in \mathcal{D}$. We suppose further that $\{\alpha_{ij}\}_{j\in\mathcal{D}}$ for a random $i \in \mathcal{R}$ can be treated as independent. Then the sum $\sum_{j\in\mathcal{D}} \alpha_{ij} \sim \mathcal{N}(\overline{S}_{\text{eff}}\mu, \overline{S}_{\text{eff}}\sigma^2)$. It follows that, for random $i \in \mathcal{R}$,

$$\gamma_i \sim \mathcal{N}\left(1-\mu\overline{X},\ \frac{\sigma^2\overline{X}^2}{\overline{S}_{\text{eff}}}\right). \tag{S18}$$

Using extreme value theory [3], and the fact that $|\mathcal{R}| \approx S$,

$$\gamma_{\max} \approx 1-\overline{X}\left(\mu-\frac{\sigma}{\sqrt{\overline{S}_{\text{eff}}}}h(S)\right), \tag{S19}$$

where $h(S)$ is a random variable[1] that scales approximately as $\sqrt{\ln S}$. In particular, $h(500) \approx 3.04 \pm 0.45$ and $h(10'000) \approx 3.85 \pm 0.35$. Using Main Text Eq. (11),

$$\gamma_{\max} \approx \frac{1-\mu-(\overline{S}_{\text{eff}}\overline{\rho}+\sqrt{\overline{S}_{\text{eff}}}h(S))\sigma}{1+(\overline{S}_{\text{eff}}-1)\mu-\overline{S}_{\text{eff}}\sigma\overline{\rho}}. \tag{S20}$$

Condition Eq. (S17) for non-invadability then amounts to

$$\sigma < \frac{\mu-1}{\overline{S}_{\text{eff}}\overline{\rho}+\sqrt{\overline{S}_{\text{eff}}}h(S)}. \tag{S21}$$

This predict lines radiating from $(\mu,\sigma) = (1,0)$. However, with $\overline{\rho} \sim (\overline{S}_{\text{eff}})^{-1/2}$, the slope of the lines are less steep for sectors with more species in the equilibrium, contrary to observation.

S5 Solution of intermittency model under unified coloured noise approximation

The unified coloured noise approximation was put forth by Jung & Hänggi [4] (see also Fox [5, 6]) to solve SDEs of the form

$$\dot{x}(t) = F(x(t)) + G(x(t))\eta(t), \tag{S22}$$

with $\eta(t)$ a coloured Gaussian noise: $\langle\eta\rangle = 0$, $\langle\eta(t)\eta(t')\rangle = \exp(-|t-t'|/\tau)$. By differentiating Eq. (S22), rearranging terms, and rescaling time into $\hat{t} = \tau^{-1/2}t$ (with $\hat{x}(\hat{t}) = x(\tau^{1/2}\hat{t})$) one obtains the exact equation

$$\frac{\mathrm{d}^2\hat{x}}{\mathrm{d}\hat{t}^2} + \frac{G'}{G}\left(\frac{\mathrm{d}\hat{x}}{\mathrm{d}\hat{t}}\right)^2 + H_\tau\frac{\mathrm{d}\hat{x}}{\mathrm{d}\hat{t}} = F + \sqrt{2}\tau^{1/4}G\hat{\xi}, \tag{S23}$$

where $\hat{\xi}$ is a standard Gaussian white noise and

$$H_\tau(x) = \tau^{-1/2} - \tau^{1/2}\left(F'(x) - \frac{G'(x)}{G(x)}F(x)\right). \tag{S24}$$

As either $\tau \to \infty$ or $\tau \to 0$, one finds $H_\tau \to \infty$, and then Eq. (S23) can be replaced by the overdamped limit

$$\frac{\mathrm{d}\hat{x}}{\mathrm{d}\hat{t}} = \frac{F}{H_\tau} + \sqrt{2}\tau^{1/4}\frac{G}{H_\tau}\hat{\xi}. \tag{S25}$$

[1] The maximum M of N i.i.d. standard Gaussian RVs tends, as $N \to \infty$, to $M = a_N + \xi/a_N$, where $a_N = \sqrt{2\ln N - \ln(4\pi\ln N)}$ and ξ follows a standard Gumbel distribution, whose mean is the Euler-Mascheroni constant (≈ 0.577) and variance is $\pi^2/6$.

It is hoped that this approximation is accurate for intermediate τ as well.

The overdamped equation Eq. (S25) can be solved for its steady state $P^*(x)$ by conventional techniques [7], e.g. under Stratonovich interpretation of the noise: the associated stationary probability current is then

$$J^* = \frac{F}{H_\tau} P^* - \tau^{1/2} \frac{G}{H_\tau} \frac{\mathrm{d}}{\mathrm{d}x} \frac{G}{H_\tau} P^*. \tag{S26}$$

In one dimension, $J^*(x)$ must be constant. Since x is a non-negative abundance in our case, we must impose a boundary at $x = 0$ through which probability cannot flow. Therefore $J^* \equiv 0$. The solution for P^* is then

$$P^*(x) \propto \exp\left\{ \int^x v(x')\,\mathrm{d}x' \right\}, \tag{S27}$$

with

$$v = (\tau^{-1/2} H_\tau) \frac{F}{G^2} + \left(\ln \frac{H_\tau}{G} \right)'. \tag{S28}$$

In the stochastic intermittency model, we have

$$F(x) = -x(k + x) + \lambda, \tag{S29}$$

$$G(x) = ux, \tag{S30}$$

$$H_\tau(x) = \tau^{-1/2} + \tau^{1/2}(x + \lambda x^{-1}). \tag{S31}$$

With these functions, the integral in Eq. (S27) can be performed exactly, yielding the result of the main article:

$$P^*(x) = \frac{1}{\mathcal{N}} e^{-[q_+(x) + q_-(\lambda/x)]} x^{-\nu} \left(\tau^{-1} + x + \frac{\lambda}{x} \right), \tag{S32}$$

$$q_\pm(y) = \frac{1}{2u^2} \left[y + (\tau^{-1} \pm k) \right]^2, \tag{S33}$$

$$\nu = 1 + \frac{k}{\tau u^2}. \tag{S34}$$

The normalization constant $\mathcal{N}$, however, we do not have in closed form; instead we calculate it numerically, after the change-of-variables $y = \ln x$, as

$$\mathcal{N} = \int_{-\infty}^{\infty} \mathrm{d}y\, e^{y(1-\nu) - q_+(e^y) - q_-(\lambda e^{-y})} \left(\tau^{-1} + e^y + \lambda e^{-y} \right). \tag{S35}$$

References

[1] J. Fraboul, "Evolution and structure emergence in complex ecological communities", PhD thesis (PSL Research University, 2023).

[2] M. Barbier and J.-F. Arnoldi, The cavity method for community ecology, bioRxiv:10.1101/147728 (2017).

[3] P. Vivo, Large deviations of the maximum of independent and identically distributed random variables, Eur. J. Phys. **36**, 055037 (2015).

[4] P. Jung and P. Hänggi, Dynamical systems: a unified colored-noise approximation, Phys. Rev. A **35**, 4464 (1987).

[5] R. F. Fox, Functional-calculus approach to stochastic differential equations, Phys. Rev. A **33**, 467 (1986).

[6] R. F. Fox, Uniform convergence to an effective Fokker-Planck equations for weakly colored noise, Phys. Rev. A **34**, 4525 (1986).

[7] C. W. Gardiner, *Stochastic methods, A handbook for the natural and social sciences* (Springer, 2009).
